# Familial Angiolipomatosis: A Report of Two Cases and Review of Clinicopathologic Features, Diagnostic Considerations, and Management Challenges in an Underserved Population

**DOI:** 10.7759/cureus.110694

**Published:** 2026-06-11

**Authors:** Aishniya Kandula, Taha Rana, Paul Bown, John Diks

**Affiliations:** 1 Pathology, Marshall University, Huntington, USA; 2 General Surgery, Marshall University Joan C. Edwards School of Medicine, Huntington, USA; 3 Pathology, Marshall University Joan C. Edwards School of Medicine, Huntington, USA

**Keywords:** angiolipoma, autosomal dominant inheritance, case series, familial angiolipomatosis, soft tissue tumor

## Abstract

Angiolipomas are benign mesenchymal tumors composed of mature adipose tissue with proliferative capillary vessels. Most cases are sporadic, presenting shortly after puberty as one or two small, painful “lumps” under the skin, followed by the appearance of two or three more each year, for several years. However, familial forms are rare and occur during the third through fifth decades of life on the trunk and extremities, with the forearms representing a common site of involvement. These lipomas generally affect consecutive generations, suggesting an autosomal dominant mode of inheritance, and are characterized by multiple, well-encapsulated, oval-to-round subcutaneous rubbery lipomas that range from a few millimeters to 25 centimeters in size and can often be misdiagnosed as neurofibromas.

We report cases of two patients who presented with multiple painful angiolipomas that involved the arms, with lesions ranging up to 5 cm in size, and multiple smaller lesions were present as well. Both patients reported pain and physical discomfort, leading to functional impairment. Excision and biopsy were performed, and histopathologic examination confirmed features consistent with angiolipoma, including vascular proliferation, mature adipocytes, and fibrin microthrombi, along with a family history supporting an autosomal dominant influence. This case report aims to highlight that familial multiple angiolipomatosis remains a poorly characterized condition, with limited data on its geographic distribution and population prevalence. Appalachia is an underrepresented region in rare disease literature and is characterized by unique demographic and healthcare access factors that may contribute to delayed diagnosis or underrecognition. This case reportprovides regional context and serves as a hypothesis-generating contribution toward understanding potential geographic patterns of this rare disorder. Early recognition is important in avoiding misdiagnosis, facilitating appropriate counseling, and guiding management.

## Introduction

Angiolipomas are benign soft-tissue tumors that typically present as painful, subcutaneous nodules, most frequently arising in young adults. Histologically, the existing literature characterizes them as benign tumors composed of mature adipose tissue interspersed with prominent capillary proliferation and characteristic fibrin microthrombi; this unique vascular component definitively distinguishes them from conventional lipomas. This paper investigates angiolipomas and a relatively uncommon subtype of adipocytic tumors with an emphasis on the inheritance patterns of familial angiolipomas [1]. While these tumors are most often sporadic, a small subset occurs in a multifocal, familial form with either autosomal dominant or recessive inheritance [2].

Because of their presentation as multiple widespread subcutaneous masses, this topic extends to the fields of dermatopathology, surgery, and clinical genetics. These familial forms are frequently misdiagnosed or missed due to their clinical resemblance to other systemic genetic disorders, such as neurofibromatosis type 1 [3, 4]. Recent molecular studies reveal distinct genetic drivers for different adipocytic tumors. Sporadic angiolipomas are primarily driven by *PIK3CA *and *PRKD2 *mutations, which promote both tumor growth and their characteristic blood vessel formation [3, 4]. In contrast, familial multiple lipomatosis (FML) - a rare condition, presenting with widespread lipomas - is associated with genetic alterations in the *HMGA2 *and *PALB2 *genes. Currently, there is a notable gap in the literature regarding the comprehensive clinical characterization, phenotypic variability, and long-term management of this specific familial variant. Consequently, accurate diagnosis requires greater clinicopathologic awareness to differentiate this entity from mimicking conditions.

These inherent diagnostic challenges may be further amplified in the Appalachian region, which faces significant healthcare access issues driven by geographic isolation, socioeconomic deprivation, and severe shortages of subspecialty providers, contributing to delayed diagnosis across multiple conditions [5, 6]. Subspecialty access has been directly linked to adverse outcomes, with the lack of specialist visits accounting for 55% of the rural-urban differences in preventable hospitalizations [7]. In regions where access to dermatopathologists or clinical geneticists is limited, a robust baseline understanding of rare soft-tissue tumors among general practitioners is critical for accurate initial triage and referral.

Ultimately, familial angiolipomatosis remains an underrecognized clinical entity that requires careful exploration to address existing knowledge gaps and to improve diagnostic accuracy, treatment planning, and longitudinal surveillance. To elucidate these features, we present case reports of two patients, with detailed clinicopathologic correlation and a comprehensive review of inheritance patterns.

## Case presentation

Case 1

A 41-year-old male presented with multiple subcutaneous masses in both upper extremities, with the largest lesion measuring 4 cm x 5 cm on the left arm (Figure 1), and smaller masses present on the right arm near the elbow. The patient reported that there was progressive enlargement of these lesions with discomfort on movement, particularly elbow flexion, with no constitutional symptoms present. 

**Figure 1:**
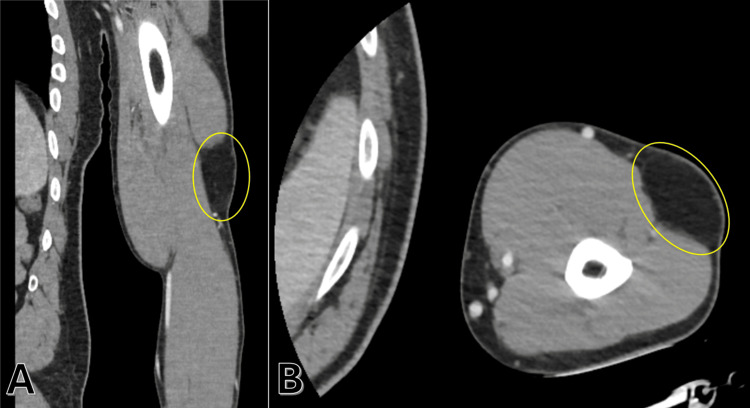
Contrast-enhanced CT scan of the left upper extremity. Coronal view (A) and sagittal view (B) demonstrating a well-circumscribed, homogeneous, low-attenuation subcutaneous mass (yellow circles) along the aspect of the left arm, consistent with a lipomatous tumor.

Given the symptomatic nature of this presentation, the patient opted to undergo surgical excision. Intraoperatively, eight lipomatous lesions were identified and excised from the left arm and eight lesions from the right arm, with most lesions being 1-2 cm in size. Gross examination showed yellow-tan, lobulated fatty tissue. Histology revealed mature adipose tissue with clusters of capillary-sized vessels containing fibrin microthrombi. The diagnosis was angiolipomas (Figure 2). 

**Figure 2:**
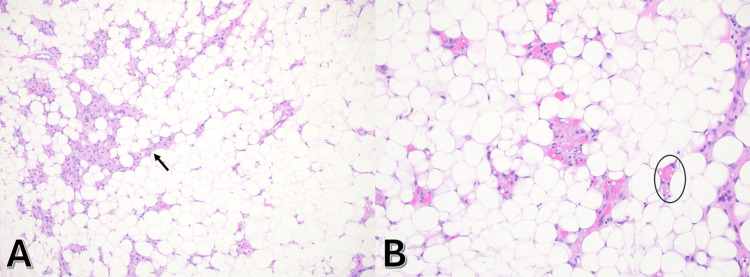
Histopathologic features of the excised left arm mass. (A) Low-power view (hematoxylin and eosin (H&E) stain, 100x) showing a well-circumscribed tumor composed of mature adipocytes admixed with a prominent, branching vascular network of capillary-sized vessels (arrow). (B) High-power view (H&E, 200x) highlighting the interspersing capillaries containing diagnostic intraluminal fibrin microthrombi (oval), characteristic of an angiolipoma. No cellular atypia or mitotic figures are identified.

Postoperatively, the patient recovered without complications, with well-healed incisions. On follow-up, he was noted to have additional lipomatous lesions involving the lower back and posterior right upper arm, associated with moderate discomfort. The patient expressed interest in the staged excision of the remaining lesions. 

Case 2

A 66-year-old female presented with numerous subcutaneous masses involving both forearms, associated with pain and functional discomfort. The lesions measured approximately 2 to 3 cm and gradually increased in number over time. The patient reported a prior excision of a similar lesion without recurrence of pain and functional discomfort. 

Family history revealed multiple relatives with similar subcutaneous tumors, though a detailed family pedigree could not be constructed due to a lack of specific relationship data. Physical examination showed numerous and difficult to count mobile masses which were soft and compressible, on her bilateral upper extremities, primarily on her forearms. The lesions exhibited a variable growth trajectory, alternating between periods of indolent, imperceptible enlargement and rapid expansion, potentially modulated by endocrine fluctuations or pharmacologic agents.

Four right forearm masses were surgically excised. Histology demonstrated well-circumscribed adipocytic lesions with prominent capillary networks and intraluminal fibrin thrombi (Figure 3). These findings were consistent with autosomal dominant familial angiolipomatosis. 

**Figure 3:**
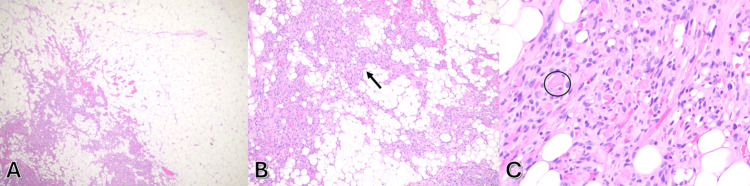
Histopathologic features of the excised forearm mass from Case 2. (A) Low-power overview (H&E, 40x) showing a well-circumscribed adipocytic lesion with prominent subcapsular/peripheral vascular proliferation tracking between mature fat lobules. (B) Medium-power view (H&E, 100x) highlighting an intimate mixture of mature adipocytes and a cellular stroma rich in proliferating, small capillary-sized channels (arrow). (C) High-power view (H&E, 400x) emphasizing the endothelial and perivascular spindle cell proliferation interspersed with capillaries containing diagnostic intraluminal fibrin microthrombi (circle).

All excised lesions across all patients and multiple surgical encounters demonstrated identical histomorphology, confirming multifocality without unexpected histologic variation. Both patients have been monitored for an average of 6 months postoperatively, demonstrating complete resolution of localized pain and no signs of recurrent lesions. A comprehensive comparison of the clinical and histopathologic features of both patients is summarized in Table 1.

**Table 1:** Clinical and histopathologic characteristics of the presented cases

Clinical Feature	Case 1	Case 2
Age and Sex	41-year-old male	66-year-old female
Primary Location	Bilateral upper extremities (arms); subsequently noted on lower back	Bilateral upper extremities (primarily forearms)
Lesion Size	Largest: 4 cm x 5 cm; Majority excised: 1-2 cm	Approximately 2 to 3 cm
Clinical Symptoms	Discomfort on movement (particularly elbow flexion); no constitutional symptoms	Pain and functional discomfort
Growth Pattern	Progressive enlargement	Variable trajectory (alternating between indolent and rapid expansion)
Family History	Not reported	Positive (multiple relatives with identical lesions)
Surgical Treatment	Excision of 16 lesions (8 from left arm, 8 from right arm)	Excision of 4 lesions (right forearm)
Histopathologic Findings	Mature adipose tissue with clusters of capillary-sized vessels containing fibrin microthrombi	Well-circumscribed mature adipocytes with prominent capillary networks and intraluminal fibrin thrombi
Diagnosis	Angiolipomatosis	Autosomal dominant familial angiolipomatosis
Follow-up (6 Months)	Complete resolution of localized pain; no recurrence of excised lesions (planned staged excision for remaining lesions)	Complete resolution of localized pain; no signs of recurrent lesions

## Discussion

Familial angiolipomatosis represents about 5 to 10 percent of angiolipoma cases. Autosomal dominant inheritance remains uncommon. Prior reports describe male predominance and upper extremity involvement. Pain often correlates with vascular components and microthrombi. Our cases mirror classic descriptions. Histologic examination demonstrates mature adipocytes containing a prominent vascular component composed of capillary-sized vessels, admixed with mature adipocytes arranged in lobules that were separated by fibrous septa. The proportion of the vascular component may vary, typically comprising 10-50% of the lesion, and tends to be more pronounced in subscapular regions. Microscopy showed the presence of intraluminal fibrin microthrombi within the capillary channels, helping distinguish angiolipomas from an ordinary lipoma. Cytologic atypia, increased mitotic activity, and features of malignancy were not identified, as lesions are benign and lack infiltrative growth patterns. These histopathologic findings assist in diagnosing angiolipoma and are important in differentiating familial angiolipomatosis from clinically similar conditions, such as neurofibromatosis type I or other adipocytic neoplasms [8]. 

Recent molecular studies have provided deeper insights into the genetic etiology distinguishing these adipocytic tumors. For instance, a significant majority of sporadic angiolipomas harbor recurrent activating *PIK3CA *mutations, suggesting that the PI3K/AKT signaling pathway is a primary driver in their pathogenesis [3]. Additionally, whole-exome sequencing has revealed that up to 80% of angiolipomas exhibit mutations in the protein kinase D2 (*PRKD2*) gene [4]. These *PRKD2 *mutations typically occur at low allele frequencies and are primarily localized within the mature adipocyte population. This supports the hypothesis that altered *PRKD2 *signaling not only initiates tumorigenesis but also promotes the prominent angiogenesis that is characteristic of these lesions [4]. Concurrently, related conditions such as familial multiple lipomatosis (FML)-a distinct, rare autosomal dominant disorder with a prevalence of approximately 1 in 50,000-manifest with widespread, non-tender, encapsulated lipomas that typically spare the head and neck, and are linked to genetic alterations in the *HMGA2 *and *PALB2 *genes [9]. Recognizing these genetic and phenotypic distinctions is crucial, as familial angiolipomatosis remains an underrecognized clinical entity. A thorough exploration of these underlying mechanisms is necessary to address existing knowledge gaps, improve diagnostic accuracy, and guide longitudinal surveillance.

Both patients showed multiple lesions limited to the subcutaneous tissue. No malignant transformation occurred. Differential diagnosis includes ordinary lipoma, neurofibromatosis type 1, and infiltrating angiolipoma. Histology distinguishes these entities. Recognition of familial disease guides counseling and long-term planning. Surgical excision remains the treatment of choice. Recurrence reflects new lesion development rather than regrowth. Both cases arise from an Appalachian patient population, a region that is historically underrepresented in the medical literature despite a high burden of chronic disease and barriers to longitudinal specialty care. The diagnosis of rare genetic conditions in rural populations faces systematic barriers at multiple levels. Rural primary care providers, even if suspicious of potential genetic conditions, face significant difficulty in pursuing genetic testing themselves and rely on referrals from specialists [10]. These referrals are followed by long waitlists and significant delays in clinical assessment, insurance clearance, and genetic testing [10, 11]. Inadequate access to subspecialty services in rural Appalachia may contribute to delayed presentation, progressive lesion burden, and staged surgical management of benign but symptomatic conditions such as multiple angiolipomatosis. Reporting cases from this population adds to the limited body of literature describing the clinical course of angiolipomas outside of large academic referral centers and underscores the importance of recognizing benign hereditary soft-tissue disorders in underserved regions. It is important to note that while the multifocal presentation and positive family history strongly suggest an autosomal dominant inheritance pattern in these cases, this remains a presumed clinical diagnosis, as formal molecular genetic testing was not performed to definitively confirm the underlying genetic etiology.

## Conclusions

Familial angiolipomatosis, while rare, requires high clinical suspicion and careful correlation of clinical and histologic findings to avoid misdiagnosis, particularly from conditions such as neurofibromatosis type I. The cases presented alongside this review exemplify these factors, portraying both the challenges of and the importance of correlating the clinical findings with the histologic features to reduce misclassifications, particularly in rural and medically underserved populations. While these cases highlight the potential for diagnostic delays in medically underserved regions, further research is required to quantify the impact of specialist access on the management of familial angiolipomatosis. Furthermore, while the multifocal presentation and positive family history are highly suggestive of an autosomal dominant inheritance pattern, this remains a clinical observation awaiting formal genetic confirmation. Areas with limited access, like the Appalachian region, to specialty care and genetic services may delay recognition. Comprehensive evaluation, including detailed family history and awareness among healthcare providers, is essential to improve diagnostic accuracy in socioeconomically disadvantaged areas, guide appropriate testing and follow-up, and optimize patient outcomes. 
